# Supplementary material for: Shared decision-making interventions in the choice of antipsychotic prescription in people living with psychosis (SHAPE): Protocol for a realist review
Source: PLoS One. 2024 Jul 25;19(7):e0304626. doi: 10.1371/journal.pone.0304626 (PMC11271866; doi:10.1371/journal.pone.0304626)
Supplement: Attachment — Submitted filename: Response to reviewers .docx [file pone.0304626.s002.docx]

Dear Dr Petkari,

Many thanks to you and your reviewers for providing positive feedback regarding our manuscript and your constructive comments. We have responded to reviewer comments below in red. Sections of the manuscript which we have subsequently amended are contained here and have been highlighted within this response for ease of identification of amendments.

Kind regards,

Ita

Reviewer #2: Thank you for the opportunity to review your work.

1. The review protocol appears to be focused on shared decision-making on antipsychotic drug treatments in the setting of psychotic illness. However, this is not clear within the aims and/or methods of the abstract. Would the authors be able to clarify this detail in their manuscript? Thank you. We have modified the methods section of our abstract to reflect the patient population our review findings will address. The methods section in the abstract (manuscript line 13-14) now reads as follows:

Methods: This review will use realist review methodology to provide a causal understanding of how and why interventions work when implementing SDM during antipsychotic treatment choices. The cohort of interest will be those experiencing psychosis where ongoing treatment with an antipsychotic is clinically indicated.

2. The inclusion of multiple stakeholder groups including clinicians and service users as well as informal and formal caregivers is a strength of the study protocol and I commend the authors for using this comprehensive approach. When engaging with stakeholder groups that include service users and informal and formal caregivers, how will the authors ensure that perspectives of these stakeholder groups are prioritized and integrated among the larger stakeholder group of clinicians and practitioners? Thank you for raising this, it is an important point that requires due consideration. Meetings of both groups will be held separately. Clinicians will meet separately to the group representing those with lived experience of psychosis and informal and formal carers. We recognised that inviting those with clinical expertise and expertise by experience to meet together could very reasonably reduce openness amongst both groups when considering factors that hinder and facilitate shared decision making, particularly during acute psychiatric care. However, the same topics will be discussed within the respective groups. Similarities and differences in interpretation of review results, next steps in programme theory development or other tasks assigned to each stakeholder group will subsequently be discussed with the other. A peer support worker representing both lived experience of psychosis and experience of working within acute psychiatric care contexts is also a member of the clinician stakeholder group to give prominence to the voice of lived experience in clinician stakeholder group meetings.

To address your important point further within the manuscript, we have modified lines 171-174. This section now reads as follows:

To ensure independent representation of the voice of both clinical expertise and expertise by experience, meetings of the clinician and lived experience stakeholder groups will meet separately. Similarities and differences in feedback from both groups will be shared with the other, and reasons for differences explored.

3. The authors identify the patient population of interest to include adult participants aged 18-65 years. Would the authors be able to clarify the rationale for exclusion of those participants over the age of 65 years? Yes, thank you. The participant population of interest to this review are:

- Inclusion:
  - Adult participants (aged 18-65) experiencing an episode of psychosis in the context of a psychotic illness where extended antipsychotic treatment is indicated.
- Exclusion:
  - Participants with treatment-resistant schizophrenia due to existence of clozapine as a preferred treatment choice amongst this cohort.
  - Participants experiencing substance/medication-induced psychosis or psychosis in the context of a general medical condition.

In our introduction section (lines 52-54), we outline ongoing suboptimal rates of shared decision-making amongst those with a severe mental illness in routine psychiatric care. Our rationale not to include adults aged >65 years relates to the average age at which a diagnosis of a severe mental illness is provided (usually between 20-30 years of age for schizophrenia, bipolar affective disorder, and major depressive disorder) to patients and thus, where antipsychotic treatment will be both started and continued. In such cases, the absence of shared decision-making will be most impactful. Other causes of psychotic symptoms more likely in those >65 years of age e.g., delirium, dementia, mild cognitive impairment are all situations where ongoing antipsychotic treatment is very unlikely to be clinically appropriate.

Additionally, whilst the absence of shared decision-making is applicable to those >65 years of age, ‘mechanisms’ which are responsible for the success of interventions and their interaction with contextual factors are likely to be different amongst younger versus older adults. For example, if a complex intervention offers patients additional information on antipsychotic treatment choices as one intervention strategy to improve the application of SDM, if increased confidence amongst patients aged <65 to engage in discussions is identified as the likely causal mechanism, it is reasonable to assume that this context-sensitive mechanism may not be equally applicable amongst someone who is 25 versus 75. Therefore, in line with the purpose of our review and intended recommendations for practice regarding implementation of SDM in SMI management, we will exclude studies solely addressing SMI in those >65 years of age. We hope this adequately addresses your query.

4. The authors report that during full-text screening that articles will be classified into high and low relevance based on previous established methods. It would be helpful to the reader to briefly explain how articles will be defined as having high or low relevance in the study. Thank you, this is useful feedback. We agree and have adapted lines 312-322 of our manuscript accordingly. This now reads as follows:

For those articles deemed to meet the inclusion criteria, IF will retrieve the full text and classify documents into high and low relevance, depending on their relevance to the programme theory, and based on established methods previously employed.^[45]^ Briefly, full-text documents will be assigned a star rating of one to five, based on a global judgement of each document’s relevance and rigour. Documents assigned a one- or two-star rating will be deemed irrelevant and not included in the review. Three-star documents will be deemed irrelevant for programme theory development but potentially useful for background material. Four-star documents will be classified as relevant for programme theory refinement. Five-star documents will be deemed the most conceptually rich or contextually thick and so, most relevant to CMOC development and programme theory refinement. All 1–3-star documents will be reviewed again before finalisation of the programme theory (ies) to confirm appropriate classification.^[32,43,44]^ A random subsample of the 10% of final documents for inclusion based on their assigned relevance and rigour judgement will be selected and assessed independently by another member of the research team (LS/EC/IM/JH/EW) to identify systematic errors.^[26, 33]^

5. How do the authors intend to evaluate rigor (i.e., trustworthiness) when completing qualitative data analysis during evidence synthesis? Many thanks for raising this. We believe from working with methodologists on this review there is no short answer to your question. Data are relevant in a realist review when they can help develop, corroborate, refute, or refine aspects of realist programme theory (or theories). The norm within realist reviews is that a programme theory that meets suggested quality criteria of simplicity, consilience, and analogy is underpinned by multiple arguments based on analysis and interpretation of many data sources, not all of which will represent empirical studies. We anticipate within this review the role of policy and regulation will be important for prescriber perceived willingness and ability to engage in shared decision-making – a key facilitator to making shared decision-making a reality within routine psychiatric settings. Thus, policy documents and texts addressing the impact of policy on service delivery and prescriber behaviour will be of high relevance. Whilst the option exists to use critical appraisal tools in the case of relevant qualitative and quantitative studies, no such tool will exist in the case of many documents relevant to the development of a coherent programme theory, for example, policy documents.

Thus, we will consider the quality of each individual data source, but, as outlined in the RAMESES guidelines, we will conceptualise assessments of quality at a more global level. Judgements about quality will be made at the levels of data, argument, and theory. To operationalise such assessment, we plan to do the following (as outlined in Wong, 2018 – see ref 44):

1. Find more than one source of data relevant to each aspect of the developing programme theory.
2. In cases of opinions etc; we will treat such data with scepticism initially until further supporting evidence is identified.
3. During building of programme theory, results will be presented initially partially, then in full, to stakeholder groups to aid in interpretation and to support development of a rigorous final programme theory.
4. We will seek to find to relevant substantive theory to support arguments made within the programme theory.
5. In cases of inadequate data or use primarily of data judged to be of poor quality to support any aspect of the programme theory, we will cautiously report such statements with appropriate qualifiers regarding the quality of data.
6. We will provide access to supporting data taken from each listed data source at the point of publication to allow for critical appraisal by peers.

We have also modified our manuscript now to address your important feedback. Lines 266-284 now read as follows:

As in other studies and in line with best practice within realist review methodology,^[25-27, 33,34]^ whilst we will consider the quality of each individual data source, we will conceptualise assessments of quality at a more global level. Judgements about quality will be made at the levels of data, argument, and programme theory. To operationalise such assessments, we plan to do the following:^[44]^

1. Find more than one source of data relevant to each aspect of the developing programme theory.
2. In cases of opinions etc; we will treat such data with scepticism initially until further supporting evidence is identified.
3. During building of programme theory, results will be presented initially partially, then in full, to stakeholder groups to aid in interpretation and to support development of a rigorous final programme theory.
4. We will seek to find to relevant substantive theory to support arguments made within the programme theory.
5. In cases of inadequate data or use primarily of data judged to be of poor quality to support any aspect of the programme theory, we will cautiously report such statements with appropriate qualifiers regarding the quality of data.
6. We will provide access to supporting data taken from each listed data source at the point of publication to allow for critical appraisal by peers.

We have also now included the following book chapter by Wong within our reference sources (ref. 44): [Data Gathering in Realist Reviews Looking for needles in haystacks | Semantic Scholar](https://www.semanticscholar.org/paper/Data-Gathering-in-RealistReviewsLooking-for-needles-Wong/a085241d87271a88b71f2094503cec14dea8707d)
